# Malaria signs, symptoms, prevention knowledge and its associated factors among rural Ethiopians

**DOI:** 10.4102/phcfm.v17i1.4885

**Published:** 2025-08-22

**Authors:** Kemal A. Kuti, Sibusiso M. Zuma

**Affiliations:** 1 Department of Health Studies, College of Human Science, University of South Africa, Pretoria, South Africa; 2 Department of Public Health, School of Health Science, Shashemene Campus, Madda Walabu University, Shashemene, Ethiopia

**Keywords:** factors, malaria, knowledge, rural community, transmission

## Abstract

**Background:**

Malaria is a leading cause of morbidity, mortality and socio-economic burden in Ethiopia. Although the country set a goal to eradicate malaria by 2030, a resurgence has been reported recently.

**Aim:**

This study was conducted to assess the signs of malaria, its symptoms and knowledge regarding prevention and its associated factors among rural Ethiopians.

**Setting:**

Three malaria-endemic rural districts in the Bale Zone, Ethiopia, constituted the setting for the study. The study respondents were household members aged 18 and older, predominantly the heads of households.

**Methods:**

A community-based cross-sectional study design was employed. Data were collected from a randomly selected 634 individuals using a pre-tested structured questionnaire. Descriptive and inferential statistics were computed using SPSS version 28.

**Results:**

Less than half of the respondents (44.2%) demonstrated a good overall understanding of the signs, symptoms and prevention of malaria, while some participants wrongly attributed malaria transmission to staying long in the sun, lack of rest and drinking alcohol. The most commonly recognised malaria symptoms include fever, headache and uncoordinated speech. Preventive measures that were widely known included eliminating mosquito breeding sites, sleeping under insecticide-treated nets and indoor residual spraying. Factors such as education, religion, marital status, family size and the presence of children and pregnant women in the household were associated with a better understanding of malaria.

**Conclusions:**

Malaria-related knowledge is low in the current study area. Some socio-demographic factors were known to have influenced malaria-related knowledge.

**Contribution:**

The study provides data on malaria-related knowledge among rural communities. The findings can be used to develop a knowledge-transfer strategy to improve communities’ knowledge and accelerate malaria elimination.

## Introduction

In 2020, there were an estimated 241 million cases and 627 000 deaths from malaria worldwide.^1^ The World Health Organization (WHO) Africa Region bore a substantial part of the malaria burden, accounting for 94% of malaria cases and 95% of malaria-related deaths in 2022. Over 80% of malaria mortality cases were among children under five years of age.^2^ Ethiopia alone accounted for 1.7% and 1.5% of global malaria cases and deaths, respectively.^2^ Moreover, a malaria resurgence has recently been observed in Ethiopia, as evidenced by a 12% increase in malaria mortalities in the year 2020,^3^ in contrast to the steady decreasing pattern seen in the previous 10 years until 2016.^4^ In 2019, the Oromia regional state experienced 9.1 deaths per 100 000 persons.^5^ The majority of districts in the Bale Zone, one of Oromia’s zones, faced a high malaria risk.

Malaria is among the leading causes of illness and death.^6,7^ In Ethiopia, 70% of the land coverage is malaria endemic, and 60 million (52%) Ethiopians are at risk of infection. Malaria transmission varies with season and geography.^8^ The peak malaria transmission is from September to December after the heavy rainy season and from April to May after the mild rainy season.^9^ Malaria has a significant medical and economic impact on humans. The greatest impact occurs among the populations of poor countries, including Ethiopia.^10^ Malaria’s impact varies according to the species composition, ecology, demographic change, socio-economic status, access to control measures and an individual’s behaviour.^11^ *Plasmodium falciparum* infection typically leads to severe forms, such as cerebral malaria, which leads to high death rates and poor quality of life.^12^ According to the Ethiopian Mini-Demographic and Health Survey (Mini-DHS) 2019, the malaria mortality rate among children aged younger than five years-old was 55/1000 live births.^13^ During pregnancy, malaria can affect both the mother and the unborn baby, leading to a high risk of low birth weight among newborn babies.^14^ In high-endemic regions, it leads to severe anaemia, spontaneous abortion, preterm delivery and foetal deaths.^15^

Malaria epidemiology is strongly related to disease information, and a lack of malaria awareness has a negative impact on malaria prevention strategies.^10,11,16,17^ Studies conducted in Africa (Zambia and Gabon) and Asia (India and Lao People’s Democratic Republic) on malaria knowledge indicate that there are misconceptions about malaria transmission among the community. Some people believe that malaria is transmitted through drinking stream water, while others believe it can be spread through walking in the rain or sexual activity. Others believe that malaria is caused by contact with malaria-infected individuals.^18,19,20,21^ Ethiopia has planned to eradicate malaria by 2030, the target date set by the WHO. As a result, the country has set an objective of eliminating malaria from 239 selected districts within six regional states.^22,23^ One critical part of this is thoroughly assessing the community’s knowledge, which is essential for planning and implementing evidence-based control measures. This study was conducted to assess malaria signs, symptoms and prevention knowledge and associated factors among communities in Bale Zone, Ethiopia.

## Methods

### Study setting and design

**FIGURE 1:**
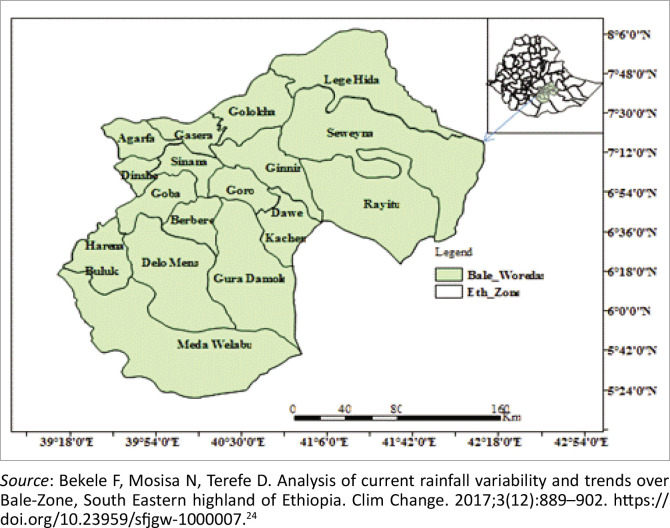
Map of the study area, Bale Zone, Ethiopia.

A community-based cross-sectional study design was used in three rural districts (Dallo Mena, Sewena, and Meda Welabu) in the Bale Zone, Oromia regional state, Ethiopia. The districts were specifically chosen for having high malaria transmission rates. The climatic conditions in the Bale Zone include 1.8% frosty highland, 7.7% highland, 25.5% midland, 63% lowland and 2% desert. Of the 18 districts in the Bale Zone, nine were pastoralist and the rest were agrarian; 14 districts are at risk of malaria transmission (Figure 1). The zone had a total population of 1 794 673 (909 164 men and 885 509 women), six hospitals, 84 health centres, 372 health posts, 100 private clinics, one NGO clinic, four other public clinics and 95 pharmacies and drug stores.

### Study population and sampling strategy

The target populations were all household (HH) members in the randomly selected Kebeles (the smallest administrative unit in Ethiopia’s context) of the three districts. Those who were permanent residents (resided there at least for 6 months) of the study area and aged 18 years and above were included in the study, while those who had a severe illness during the data-collection period or a known mental disorder that affects the accuracy of the given data were excluded. The total sample size was 634. It was calculated using a single population proportion formula based on the following assumptions:

- The estimated proportion (*P*) of good knowledge on malaria signs, symptoms and prevention was 50%.
- *Z*_α__/2_ = confidence level taken as 95% = 1.96
- A margin of error (*d*) of 5%
- A designing effect of (*k*) of 1.5
- A non-response rate of 10%

Therefore, *n*_0_ = (Z_α__/2_)^2^ * (p*q)/*d*^2^ = (1.96*1.96)*(0.5*0.5)/(0.05*.05) = 384

Considering a designing effect (*k* = 1.5), *n*_1_ = *n*_0_*k = 384*1.5 = 576.

Finally, considering the non-response rate of 10%, ***n***_**f**_ = (*n*_1_) + (*n*_1_*0.1) = 576 + 58 = **634.**

Where, *n*_0_ was the initial sample size,

*n*_1_ was the sample size calculated in consideration of the design effect (*k*).

*n*_f_ was the final sample size, including the non-response rate.

**FIGURE 2:**
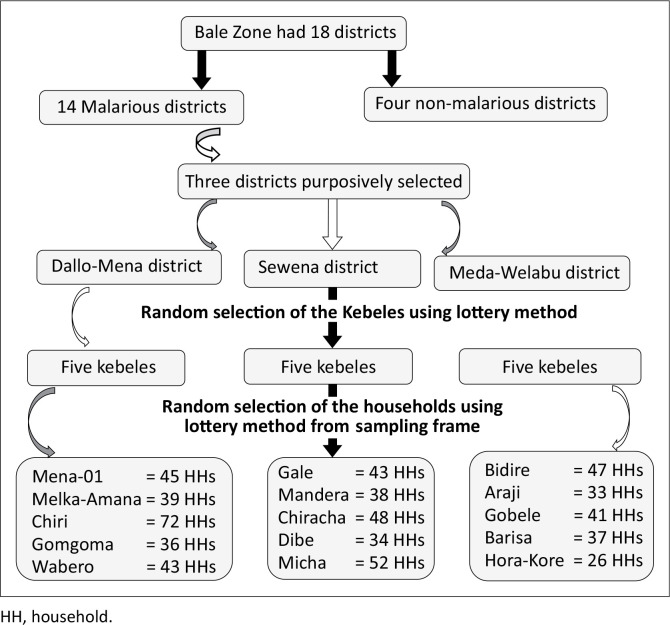
Sampling procedure, Bale Zone, Ethiopia.

Initially, three districts were purposively selected out of 14 malarious districts in the Bale Zone. This was followed by a random selection (lottery method) of five Kebeles from each selected district. Finally, eligible households were selected by a simple random sampling technique using the lottery method from a sampling frame (Figure 2).

The heads of the selected households were then interviewed. If the head of the family was not at home during the visit, their partner or a family member aged 18 or older, was interviewed instead.

### Data-collection instrument

The data were collected using a structured interviewer-administered questionnaire, which was developed by reviewing the literature from previous studies in consideration of the objectives and research questions. The questionnaire was initially developed in English, translated to Afan Oromo (the local language) and then back translated to English in order to ensure consistency between the two versions. It covered contents such as socio-demographic characteristics, economic condition, history of illness and knowledge about malaria.

The questionnaire was designed with simple instructions that improved its clarity, conciseness and logical flow while reducing ambiguity, repetition and the inclusion of extraneous items. To reduce coding errors, all possible responses to questions were pre-coded with ‘other specify’ as an option, except for continuous variables such as age, monthly income and family size, where open-ended questions were used.

Prior to data collection, the questionnaire was pretested on 5% (*n* = 32) of the sample size from the non-sampled kebele. The pre-test was useful in determining the feasibility of the actual study, the clarity of the questions and the timing of questionnaire completion. The questionnaire was also submitted to relevant specialists in the subject, and it was finalised after required changes were made to incorporate all opinions received during the process.

To ensure high-quality results, the questionnaire’s validity and reliability were assessed. We used exploratory factor analysis (EFA) with the assistance of a statistician to confirm the construct validity of each item. To investigate the relationships between different questionnaire items, the related items were aggregated into a factor. The Keiser–Meyer–Olkin (KMO) Measure of Sampling Adequacy (MSA) criterion was used to determine the sampling adequacy of the malaria knowledge assessment questions.

The overall KMO-MSA was 0.767 for 17 items (Table 1). This value was higher than the minimum acceptable level (0.600) and ensured that the sample of items was adequate. The value for the determinants was 0.007, and that of the Bartlett’s test of sphericity (BTS) was *χ*^2^ = 3089.541.

**TABLE 1:** Statistical validity and Keiser–Meyer–Olkin-Measure of Sampling Adequacy criterion (knowledge items), Bale Zone, Ethiopia.

Dimensions	Items	Number of items	KMO
Knowledge of malaria symptoms	Hotness of the body as a symptom of malaria infection Darkness of urine as a symptom of malaria infection Joint pain as a symptom of malaria infection Headache as a symptom of malaria infection Disorientation/talking nonsense as a symptom of malaria infection Weakness/dizziness as a symptom of malaria infection Difficulty in breathing as a symptom of malaria infection Coma as a symptom of malaria infection	8	0.84
Knowledge of malaria causes	Drinking too much alcohol as cause of malaria Not resting enough or a lack of sleep as cause of malaria Staying long in the Sun as cause of malaria	3	0.55
Knowledge of malaria prevention and control	Taking medication/prophylaxis as a malaria-prevention method Destroying breeding sites of mosquitoes as a malaria-prevention method Sleeping under ITN for malaria prevention Use of mosquito repellent cream as a malaria-prevention method Use of the IRS as a malaria-prevention method Seeking health care immediately after malaria infection is considered as malaria prevention	6	0.71
Overall scale reliability		17	0.77

ITN, insecticide-treated bed net; IRS, indoor residual sprays; KMO, Keiser–Meyer–Olkin.

The Cronbach’s alpha coefficient was calculated to assess the extent to which research respondents could deliver identical responses to the same set of questions asked several times under the same conditions (reliability). The predetermined cutoff for the Cronbach’s alpha coefficient value was 0.70. In the current study, the Cronbach’s alpha coefficient value for 17 items used to measure the knowledge level was 0.779. Of the 17 items, 12 items that explained 89.4% of the variance in the principal component analysis (PCA) were used for composite index construction (Table 2).

**TABLE 2:** Scale reliability for knowledge items, Bale Zone, Ethiopia.

Dimensions	Items	Number of items	Cronbach’s alpha
Knowledge of malaria symptoms	Hotness of the body as a symptom of malaria infection Darkness of urine as a symptom of malaria infection Joint pain as a symptom of malaria infection Headache as a symptom of malaria infection Disorientation/uncoordinated speech as a symptom of malaria infection Weakness/Dizziness as a symptom of malaria infection Difficulty in breathing as a symptom of malaria infection Coma as a symptom of malaria infection	8	0.78
Knowledge of malaria causes	Drinking too much alcohol as a cause of malaria infection Not resting enough or a lack of sleep as a cause of malaria Staying long in the Sun as a cause of malaria infection	3	0.50
Knowledge of malaria prevention	Taking medication/prophylaxis as a malaria-prevention method Destroying breeding sites of mosquitoes as a malaria prevention Sleeping under an ITN as a malaria prevention method Use of mosquito repellent cream as malaria prevention Use of indoor residual spray as malaria prevention Seeking health care immediately after malaria infection is suspected as a malaria prevention method	6	0.63
Overall scale reliability		-	0.78

ITN, insecticide-treated bed net.

### Data-collection process

Six data collectors were recruited to conduct quantitative data collection. They were master’s degree holders in public health and nursing professionals working in public universities. They had four to seven years of experience conducting research activities and data collection in similar settings.

After the final version of the questionnaire was formatted and all logistics were made ready, the actual data collection took place from 05 March 2018 to 25 March 2018, using an interviewer-administered questionnaire.

### Data analysis

Each complete questionnaire was checked item by item to ensure that the data were error-free and complete. The data were entered in the Statistical Package for Social Science (SPSS) version 28. Each item used to assess knowledge was dichotomised and coded ‘0’ for incorrect and ‘1’ for correct responses. To compute the overall knowledge score, a composite index was constructed based on the PCA. Descriptive statistics such as mean, proportions and percentages were calculated. Inferential statistics including crude and adjusted odds ratios with 95% confidence intervals were computed using binary logistic regression to investigate a relationship between the dependent variable (knowledge level) and independent variables including sociodemographic characteristics (residence, age, gender, marital status, ethnicity, religion, educational status and occupation), history of malaria infection (current illness and ever contracting malaria) and family information (family size, presence of children younger than five years-old and pregnant women).

The model fit was checked using the Hosmer–Lemshow goodness-of-fit test, and multicollinearity was checked using the variance inflation factor (VIF) and tolerance. Nine variables that showed a significant association with knowledge level at a *p*-value of less than 0.05 during bivariable logistic regression analysis were included in the multivariable logistic regression model. The presence of an association was declared at a *p*-value of less than 0.05.

### Ethical considerations

Ethical approval and an ethical clearance certificate (REC-012714-039) were obtained from the higher degree committee of the University of South Africa (Unisa). A letter of permission was obtained from the Oromia regional health bureau and communicated to the zonal health department and district health offices. The respondents were informed about the study’s purpose, benefits, potential effects (if any), their freedom to participate or not and privacy and confidentiality concerns, and written consent was obtained. To ensure anonymity, study respondents were interviewed at their residences. In terms of information confidentiality, no identifiers of the respondents’ identities were recorded; also, all data were securely stored to prevent unauthorised access.

## Results

### Socio-demographic characteristics

The study had a total of 634 respondents. The study respondents’ mean age was 35.3 years, with an 8-year standard deviation (s.d.). As shown in Table 3, most (40.7%; *n* = 258) of the study respondents were in the age group of 31–40 years; more than half were females (55.8%; *n* = 354) and about three-quarters (76.3%; *n* = 484) were currently married. Slightly more than half (52.7%, *n* = 334) did not attend formal education. About 59.6% (*n* = 378) and 65.9% (*n* = 418) of the households had children aged below 5 years and pregnant women, respectively. Nearly four-fifths (79.2%; *n* = 502) were Oromo by ethnicity, 76.3% (*n* = 484) were Muslims, 45.1% (*n* = 286) were farmers and 13.23% (*n* = 60) earn a monthly income of only 150–1000 Ethiopian birr (Table 3).

**TABLE 3:** Socio-demographic characteristics of the respondents (*N* = 634), Bale Zone, Ethiopia.

Variables	Count (*n*)	Proportion (%)
**Age (years)**		
19–30	204	32.20
31–40	258	40.70
> 40	172	27.10
**Gender**		
Male	280	44.20
Female	354	55.80
**Educational status**		
Cannot read a write	204	32.20
Read and write only	130	20.50
Primary Grades (1–8)	180	28.40
Secondary and above	120	18.90
**Marital status**		
Single	54	8.50
Married	484	76.30
Widowed	42	6.60
Divorced	54	8.50
**Children in househould younger than five years-old**		
No	256	40.40
Yes	378	59.60
**Pregnant women in the HH**		
No	216	34.10
Yes	418	65.90
**Religion**		
Muslim	204	32.20
orthodox	258	40.70
Protestant	172	27.10
**Ethnicity**		
Oromo	280	44.20
Amhara	354	55.80
Others	54	8.50
**Occupation**		
Farmer	286	45.10
Civil servants and students	108	17.00
Merchants	240	37.90
**Family monthly income (currency ETB) (*N* = 454)**		
150–1000	60	13.23
1001–2000	84	18.50
2001–3000	130	28.63
> 3000	180	39.64

HH, household; ETB, Ethiopian birr.

### Knowledge of the cause and signs and symptoms of malaria

The study respondents demonstrated good knowledge of the cause of malaria, with the exception of a few misunderstandings. All study respondents attribute malaria transmission to mosquito bites. However, in addition to the mosquito bite, about 5.7% (*n* = 36), 2.8% (*n* = 18) and 0.9% (*n* = 6) attributed malaria transmission to staying long in the sun, lack of rest or not sleeping enough and drinking too much alcohol, respectively (Table 4).

**TABLE 4:** Knowledge on cause and signs and symptoms of malaria (*N* = 634), Bale Zone, Ethiopia.

SN	Variables	Response	*n*	%
**How do individuals acquire malaria?**				
1	Staying long in the Sun	Yes	36	5.7
2	Lack of rest or not sleeping enough	Yes	18	2.8
3	Drinking too much alcohol	Yes	6	0.9
4	Mosquito bite	Yes	100	100.0
**What are the common signs and symptoms of malaria?**				
1	Fever or increased body temperature	Yes	604	95.3
2	Headache	Yes	502	79.2
3	Uncoordinated speech or disorientation	Yes	342	53.9
4	Joint pain	Yes	226	35.6
5	Weakness or dizziness	Yes	264	41.8
6	Dark urine	Yes	76	12.0
7	Difficult breathing	Yes	184	29.0
8	Coma or loss of consciousness	Yes	262	41.3

SN, serial number.

Unlike the cause of malaria, most study respondents lacked knowledge of most of the signs and symptoms of malaria. Of the eight common signs and symptoms of malaria presented to the study respondents, only three were known by more than 50% of the respondents, which indicates a lower level of understanding in this regard (Table 4).

### Knowledge of malaria prevention methods

**FIGURE 3:**
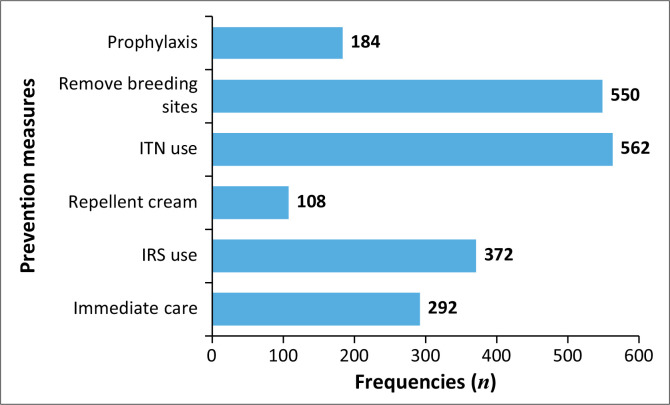
Respondents’ knowledge on malaria prevention Methods or techniques (*N* = 634), Bale Zone, Ethiopia.

Malaria prevention methods relatively known by the majority (more than 50% of the respondents) include destroying malaria breeding sites 86.8% (*n* = 550), sleeping under insecticide-treated bed nets (ITNs) 88.6% (*n* = 562) and using indoor residual sprays (IRS) 58.7% (*n* = 372), while the lesser-known prevention methods include the use of prophylactic medications (29% *n* = 184), mosquito repellent cream (16.7% *n* = 108) and immediate health seeking for suspected malaria cases 46.1% (*n* = 292) (see Figure 3).

### Overall malaria knowledge

The overall knowledge was measured based on the composite index that was constructed from 17 items used to assess the respondents’ knowledge on the symptoms of malaria, the causes of malaria and the method of malaria prevention after performing PCA. Slightly less than half (44.2%, *n* = 280) had good knowledge of malaria; the rest, 54.8% (*n* = 354), had poor knowledge.

#### Factors associated with the level of knowledge

Educational level, religion, marital status, family size, the presence of children under the age of 5 and pregnant women in the household all had a substantial impact on knowledge of malaria. However, gender, family monthly income and malaria infection among household members did not show a significant association with the dependent variable in the current study (Table 5).

**TABLE 5:** Factors associated with the level of knowledge, Bale Zone, Ethiopia.

SN	Variables	Response	Knowledge		COR	95%	CI	AOR	95%	CI	*p*
			Poor	Good							
1	Gender	Male	138	142	1.61	1.17	2.21	1.25	0.74	2.13	0.410
		Female	216	38	1.00	-	-	1.00	-	-	-
2	Education	Unable to read and write	138	66	1.00	-	-	1.00	-	-	-
		Able to read and write	108	22	0.43	0.25	0.73	0.60	0.25	1.43	0.250
		Primary (1–8 grades)	72	108	3.14	2.06	4.77	3.25	1.55	6.82	0.002
		Secondary and above	36	84	4.88	2.99	7.96	9.55	3.91	23.31	< 0.001
3	Religion	Muslim	246	238	1.00	-	-	1.00	-	-	-
		Orthodox Christian	102	30	0.30	0.20	0.47	0.36	0.20	0.68	0.002
		Protestant	6	12	2.07	0.76	6.00	0.99	0.21	4.67	0.992
4	Marital status	Single	24	30	2.50	1.15	5.50	3.03	0.81	11.33	0.099
		Married	270	214	1.59	0.88	2.78	0.78	0.27	2.26	0.642
		Divorced	24	18	1.50	0.65	3.45	5.91	1.42	24.60	0.015
		Widowed	36	18	1.00	-	-	1.00	-	-	-
5	Children (younger than five years-old) in the household	No	234	144	1.00	-		1.00	-	-	-
		Yes	120	136	1.84	1.34	2.54	3.32	1.82	6.06	< 0.001
6	Pregnant in the HHs	No	246	172	1.00	-		1.00	-	-	-
		Yes	108	108	1.43	1.03	2.00	2.84	1.58	5.10	< 0.001
7	Malaria ever among HH members	No	174	166	1.51	1.10	2.08	1.57	0.93	2.65	0.092
		Yes	180	114	1.00	-	-	1.00	-	-	-
8	Family size	1–5	150	138	1.00	-	-	1.00	-	-	-
		6–10	180	112	0.68	0.49	0.94	2.03	1.17	3.51	0.012
		11–15	24	30	1.36	0.76	2.44	19.82	5.53	70.92	< 0.001
9	Family monthly income (currency ETB)	150–1000	42	18	1.00	-	-	1.00	-		-
		1001–2000	60	24	0.93	0.45	1.93	0.55	0.19	1.61	0.277
		2001–3000	60	70	2.72	1.42	5.22	2.44	0.98	6.09	0.055
		> 3000	78	102	3.05	1.63	5.71	1.42	0.62	3.26	0.405

HH, household; AOR, adjusted odds ratio; COR, crude odds ratio; SN, serial number; ETB, Ethopian birr.

Individuals who had attended primary school and secondary school and above were 3.25 (adjusted odds ratio [AOR]: 3.25, 95% CI: 1.55–6.72) and 9.55 (AOR: 9.55, 95% CI: 3.91–23.31) times more knowledgeable, respectively, than those who were unable to read and write. Orthodox Christians were 64% less likely to have good knowledge of malaria compared to their Muslim counterparts (AOR: 0.36, 95% CI: 0.02–0.68), while no statistically significant difference was observed between Muslims and Protestants.

Divorced individuals were 5.91 times more likely to be knowledgeable compared to widowed ones (AOR: 5.91, 95% CI: 1.42–24.60), while no significant difference was observed between single, married and divorced individuals. People from households with children aged below 5 years were 3.32 times more knowledgeable than their counterparts (AOR: 3.32, 95% CI: 1.82–6.06).

People from households with pregnant women were 2.84 times more knowledgeable than their counterparts (AOR: 2.84, 95% CI: 1.58–5.10). Those from households with family sizes 6–10 and 11–15 were 2.03 (AOR: 2.03, 95% CI: 1.17–3.51) and 19.82 (AOR: 19.82, 95% CI: 5.53–70.92) times more knowledgeable compared to those with 1–5 sized families (Table 5).

## Discussion

The current study found that people had inadequate knowledge about malaria signs and symptoms, as well as how to prevent them. Only 44.2% of research respondents had an adequate understanding. Education, religion, marital status, family size and the presence of children and pregnant women in the household all have a substantial impact on knowledge.

### Knowledge of malaria cause

In the current study, all respondents attributed malaria transmission to mosquito bites, while some study respondents additionally attributed it to staying long in the sun, lack of rest or not sleeping enough and drinking too much alcohol. In agreement with this study, studies conducted in Gabon, India and Cameroon revealed that the majority attributed the cause of malaria to mosquito bites.^19,20,25^ Studies conducted in southern Africa, Gabon and India also disclosed the presence of individuals who think malaria spreads by eating stale or bad food, fresh fruit, maize or sugarcane, or by drinking contaminated water.^19,20,26^

The current study revealed that water-containing pits, the bushes in the home environment, poor environmental sanitation and unsanitary human actions, including poor liquid waste and latrine management, were sources of malaria transmission. Other studies from Cameroon and India also reported that stagnant or dirty water, gutters, muddy sludge and puddles were sources of malaria transmission.^20,25^ This means that the community is knowledgeable about malaria causes; however, their knowledge about the situations that facilitate malaria transmission lacks consistency. This may negatively affect the prevention practices; thus, a comprehensive community-based knowledge transfer intervention is very crucial.^26^

In contrast to the current study, another study conducted in rural Zambia reported drinking dirty water and touching a malaria patient as routes of malaria transmission.^21^ The possible explanation for the observed difference may be because of the difference between the two studies, including setting (Ethiopia vs. Zambia), the sample size (634 vs. 75) and the scope of the study (knowledge assessment vs. knowledge, attitude and practice [KAP] survey). Unlike other studies, the current study did not report the misconception that malaria can be transmitted by walking in the rain or through sexual contact.^19,20^ The possible explanations include differences in study respondents, setting and scope of the studies. The current study was conducted in three rural districts, focusing on the assessment of knowledge of signs and symptoms, causes and prevention methods among the general population. However, the study in India was conducted in an urban setting, focusing on KAP.^20^ Furthermore, the respondents of the Gabon study were reproductive-age women, and the focus of the study was KAP on prevention.^19^

These findings clarified the presence of misconceptions about malaria that should be considered, which calls for the design of a contextual intervention strategy and effective implementation for a better understanding of the community at the grassroots level.

### Knowledge of sign and symptoms of malaria

The current study revealed that the community had limited knowledge of the signs and symptoms of malaria. Only three signs and symptoms were known by more than 50% of the respondents, which include fever (95.3%), headache (79.2%) and talking nonsense (53.9%). The studies that agreed with the current study also revealed that fever, headache, vomiting and appetite loss are the most common malaria symptoms known by the majority of the respondents.^20,26,27^ On the other hand, symptoms like pain, backache, fatigue, vomiting and anorexia that were reported by other studies^26,27^ were not reported by the current study. The difference may be because the current study differs from others in terms of study setting (community-based) and the sample size (634 households). The study conducted in southern Africa was multinational (Zimbabwe and Zambia)^26^ and had a large sample size (2066 households), while that of Gonder (Ethiopia) was facility-based with a smaller sample size (390).^27^

In general, even though the community is aware of a few very common symptoms, their knowledge of malaria symptoms is limited. Thus, detailed gap identification and appropriate intervention are necessary to overcome the associated consequences.

### Knowledge of malaria prevention methods

The majority of the survey respondents mentioned destroying malaria-breeding sites, sleeping under ITNs and using IRS as malaria prevention methods. However, only a few respondents mentioned the use of prophylactic medications, mosquito repellent cream and immediate health-seeking for suspected malaria cases. The study conducted in South Gondor also revealed that the majority of the respondents mentioned using ITNs and IRS, while a few individuals mentioned fumigation.^28^ Furthermore, the study conducted in Tanzania revealed that the majority of the study respondents mentioned ITN use, IRS and destroying breeding sites as measures of malaria prevention.^29^ These findings suggest a gap in awareness or access to other effective prevention strategies, such as prophylactic medications and timely health-seeking behaviours. Public health initiatives must address these gaps, ensuring that communities are aware of all available techniques for efficiently combating malaria.

In contrast, neither of the two studies reported other malaria-prevention measures such as the use of prophylactic medications, the use of a mosquito repellent cream or immediate health seeking for suspected malaria cases.^28,29^ The difference may be because of the variation in the study setting, design and time frame. The findings imply that the community in the current study area has demonstrated moderately good knowledge on malaria prevention measures, even if the commonly known measures are few. Thus, additional efforts are required to improve.

### Overall malaria knowledge

Malaria awareness is low in the current study area. The majority of the survey respondents (54.8%) lacked knowledge. This finding is consistent with the findings of a study conducted in Cambodia,^30^ which found that the community’s understanding of malaria epidemiology and vector ecology was limited. This could be attributed to the lower literacy levels seen in the two research populations.

On the other hand, the findings of the current study are lower than the results of other studies conducted in southern Africa and Cameroon.^26,31^ The difference may be because of the variation in the characteristics of the population, like social structure, economic condition and level of literacy. The other possible explanation could be methodological differences between the current study and other studies. This implies that the overall knowledge in the study area is low, and this calls for improvements in information, education and communication to enhance the knowledge of the community. As pointed out by others, to prevent malaria infection and promote malaria-free zones, understanding the community’s knowledge of malaria control is essential.^29^ It is crucial that educational initiatives focus primarily on best practices to increase local populations’ adherence to malaria-prevention initiatives.^31^

### Factors associated with malaria knowledge

The present study shows that those persons who completed elementary school and secondary school or higher possessed knowledge levels 3.25 and 9.55 times more, respectively, than those who were illiterate. This finding aligns with the study conducted in South Gonder, Ethiopia, which indicated that the highest knowledge scores were observed among persons with college education and above (84.3%) and those with high school education (82.9%) compared to uneducated individuals.^28^ Another institutional-based cross-sectional study conducted at Adis Zemen Hospital, Northwestern Ethiopia, also revealed that those who attained primary education were more knowledgeable than those who were unable to read and write.^30^ This implies that awareness creation and knowledge transfer activities should primarily target people with no formal education and those with lower educational levels to improve their knowledge of malaria and its prevention.

Orthodox Christians were 64% less likely to have good knowledge of malaria compared to their Muslim counterparts, while no statistically significant difference was observed between Muslims and Protestants. Divorced individuals were 5.91 times more likely to be knowledgeable compared to widowed individuals, while no significant difference was observed between single, married and divorced individuals. Contrary to the current study, a cross-sectional household survey conducted in Nyabondo, Western Kenya, reported that marital status and religion were not significantly associated with knowledge related to malaria.^32^ The possible explanation for the observed variation could be because of the differences between the two studies in terms of the sample size and the type of data. The current study utilised quantitative data from 634 households, while the other study utilised both qualitative and quantitative data from 80 households.

People from households with children younger than five years-old were 3.32 times more knowledgeable compared to their counterparts; people from households with pregnant women were 2.84 times more knowledgeable compared to their counterparts. Those from households sizes six to 10 family members, and 11–15 family members were 2.03 and 19.82 times more knowledgeable compared to those with one to five family members. This finding suggests that larger families and those with young children or pregnant women are more engaged in acquiring knowledge, possibly because of the heightened need for information related to child-rearing and prenatal care. Consequently, targeted educational resources may be beneficial in further enhancing awareness and understanding among smaller families.

The current study contradicts previous research that found statistically significant relationships between malaria knowledge and other factors such as age, gender, family monthly income, place of residence and occupation.^28^ The disparities may be because of the differences in the study population and the scope of the studies. The respondents of the current study were all adults, while those of the other study were adult men only. Furthermore, the current study specifically focused on knowledge assessment, unlike other studies that assessed knowledge, attitude and practice of malaria control.

## Strengths and limitations

### Strengths

The existing literature was used to inform the construction of the data-collection tool, which ensured its reliability and statistical validity. During the data-collection period, repeated visits to households ensured the highest response rate. Furthermore, the study produced very informative findings on malaria signs, symptoms and preventive knowledge in rural areas.

### Limitations

Because of the cross-sectional character of this study, determining a cause–effect relationship is difficult. Furthermore, the knowledge assessment primarily focused on three aspects: knowledge of malaria causes, signs and symptoms, and prevention methods, with little emphasis placed on sources of information and malaria definition. However, these elements were considered for further qualitative investigation.

## Conclusion

Malaria knowledge is limited in the current study area. Knowledge of malaria signs and symptoms, as well as prevention methods, was quite limited, despite a thorough grasp of the cause of malaria. Sociodemographic characteristics such as education level, religion, marital status and family size all had an impact on malaria knowledge. Furthermore, having pregnant women and children younger than five years-old in the household was linked to malaria knowledge. Identification of the community’s malaria control knowledge is critical for malaria prevention and the establishment of malaria-free zones.

## Recommendations

We recommend a meticulous design and execution of a contextualised knowledge transfer strategy to enhance the community’s understanding of malaria and expedite the journey towards malaria elimination. Subsequent research should concentrate on an effective knowledge transmission technique suitable for rural contexts.

## References

[1] Centers for Disease Control and Prevention. Malaria impact worldwide [homepage on the Internet]. Centers for CDC; 2021 [cited 2022 July 27]. Available from: https://www.cdc.gov/malaria/malaria_worldwide/impact.html

[2] World Health Organization. World malaria report 2023 [homepage on the Internet]. 2023 [cited 2023 Dec 08]. Available from: https://www.who.int/news-room/fact-sheets/detail/malaria

[3] World Health Organization. World malaria report 2021. World Health Organization; 2022 [cited 2022 July 30]. Available from: https://reliefweb.int/report/world/world-malaria-report-2021

[4] Jemal A, Ketema T. A declining pattern of malaria prevalence in Asendabo Health Center Jimma zone, Southwest Ethiopia. BMC Res Notes. 2019;12:1–5. 10.1186/s13104-019-4329-6 31133048 PMC6537395

[5] National Data Management Center for Health. Trend and burden of malaria in Ethiopia [homepage on the Internet]. National Data Management Center for Health. 2022 [cited 2024 Jan 22]. Available from: https://ndmc.ephi.gov.et/download/trend-and-burden-of-malaria-in-ethiopia/

[6] Kendie FA, Hailegebriel W/kiros T, Nibret Semegn E, Ferede MW. Prevalence of malaria among adults in Ethiopia: A systematic review and meta-analysis. J Trop Med. 2021;2021(1):8863002. 10.1155/2021/8863002 33747096 PMC7952180

[7] Memvanga PB, Nkanga CI. Liposomes for malaria management: The evolution from 1980 to 2020. Malar J. 2021;20(1):327. 10.1186/s12936-021-03858-0 34315484 PMC8313885

[8] Ketema T, Bacha K, Getahun K, Portillo HA, Bassat Q. Plasmodium vivax epidemiology in Ethiopia 2000-2020: A systematic review and meta-analysis. PLoS Negl Trop Dis. 2021;15(9):e0009781. 10.1371/journal.pntd.0009781 34525091 PMC8476039

[9] Tefera S, Bekele T, Getahun K, Negash A, Ketema T. The changing malaria trend and control efforts in Oromia special zone, Amhara regional state, North-East Ethiopia. Malar J. 2022;21(1):128. 10.1186/s12936-022-04149-y 35459176 PMC9034650

[10] Rudasingwa G, Cho SI. Determinants of the persistence of malaria in Rwanda. Malar J. 2020;19:1–9. 10.1186/s12936-020-3117-z 31964371 PMC6975052

[11] Fornace KM, Diaz AV, Lines J, Drakeley CJ. Achieving global malaria eradication in changing landscapes. Malar J. 2021;20(1):69. 10.1186/s12936-021-03599-0 33530995 PMC7856737

[12] Schiess N, Villabona-Rueda A, Cottier KE, Huether K, Chipeta J, Stins MF. Pathophysiology and neurologic sequelae of cerebral malaria. Malar J. 2020;19:1–2. 10.1186/s12936-020-03336-z 32703204 PMC7376930

[13] Initiative UP. Malaria operational plan FY 2020. Washington, DC: US President’s Malaria Initiative. 2020.

[14] Bakken L, Iversen PO. The impact of malaria during pregnancy on low birth weight in East-Africa: A topical review. Malar J. 2021;20:348 34429121 10.1186/s12936-021-03883-z PMC8386002

[15] Romero M, Leiba E, Carrión-Nessi FS, et al. Malaria in pregnancy complications in Southern Venezuela. Malar J. 2021;20:1–8. 10.1186/s12936-021-03728-9 33858446 PMC8051027

[16] Fikrie A, Kayamo M, Bekele H. Malaria prevention practices and associated factors among households of Hawassa City Administration, Southern Ethiopia, 2020. PloS One. 2021;16(5):e0250981. 10.1371/journal.pone.0250981 33984022 PMC8118284

[17] Tarekegn M, Tekie H, Dugassa S, Wolde-Hawariat Y. Malaria prevalence and associated risk factors in Dembiya district, North-western Ethiopia. Malar J. 2021;20(1):372. 10.1186/s12936-021-03906-9 34535130 PMC8447688

[18] Vilay P, Nonaka D, Senamonty P, et al. Malaria prevalence, knowledge, perception, preventive and treatment behavior among military in Champasak and Attapeu provinces, Lao PDR: A mixed methods study. Trop Med Health. 2019;47:1–2. 10.1186/s41182-019-0138-9 30700970 PMC6347756

[19] Imboumy-Limoukou RK, Maghendji-Nzondo S, Sir-Ondo-Enguier PN, et al. Malaria in children and women of childbearing age: Infection prevalence, knowledge and use of malaria prevention tools in the province of Nyanga, Gabon. Malar J. 2020;19(1):1–8. 10.1186/s12936-020-03411-5 33138819 PMC7607695

[20] Rajvanshi H, Saha KB, Sharma RK, et al. Assessing community knowledge, attitude and practices to strengthen communication strategy for malaria elimination demonstration project in Mandla. Malar J. 2021;20(1):354. 10.1186/s12936-021-03884-y 34454483 PMC8403442

[21] Jumbam DT, Stevenson JC, Matoba J, et al. Knowledge, attitudes and practices assessment of malaria interventions in rural Zambia. BMC Public Health. 2020;20(1):1–5. 10.1186/s12889-020-8235-6 32050923 PMC7017631

[22] Initiative PM. President’s malaria initiative Ethiopia. Malaria Operational Plan FY [homepage on the Internet]. Center for Communicable Diseases Control; 2017 [cited n.d.]. https://reliefweb.int/report/ethiopia/president-s-malaria-initiative-ethiopia-malaria-operational-plan-fy-2019

[23] Presidents Malaria Initiative. President’s Malaria Initiative Ethiopia Malaria Operational Plan FY. Center for Communicable Diseases Control; 2019.

[24] Bekele F, Mosisa N, Terefe D. Analysis of current rainfall variability and trends over Bale-Zone, South Eastern highland of Ethiopia. Clim Change. 2017;3(12):889–902.

[25] Talipouo A, Ngadjeu CS, Doumbe-Belisse P, et al. Malaria prevention in the city of Yaoundé: Knowledge and practices of urban dwellers. Malar J. 2019;18(1):1–3. 10.1186/s12936-019-2799-6 31072344 PMC6509831

[26] Kanyangarara M, Hamapumbu H, Mamini E, et al. Malaria knowledge and bed net use in three transmission settings in southern Africa. Malar J. 2018;17(1):1–2. 10.1186/s12936-018-2178-8 29351795 PMC5775538

[27] Flatie BT, Munshea A. Knowledge, attitude, and practice towards malaria among people attending Mekaneeyesus Primary Hospital, South Gondar, Northwestern Ethiopia: A cross-sectional study. J Parasitol Res. 2021;2021(1):5580715. 10.1155/2021/5580715 34976405 PMC8718288

[28] Yasuoka J, Kikuchi K, Nanishi K, et al. Malaria knowledge, preventive actions, and treatment-seeking behavior among ethnic minorities in Ratanakiri Province, Cambodia: A community-based cross-sectional survey. BMC Public Health. 2018;18:1206. 10.1186/s12889-018-6123-0 30367615 PMC6203989

[29] Yitayal Ayalew Goshu YA, Azeb Ewinetu Yitayew AE. Malaria knowledge and its associated factors among pregnant women attending antenatal clinic of Adis Zemen Hospital, North-western Ethiopia, 2018. PLoS One. 2019;14(1):e0210221. 10.1371/journal.pone.0210221 PMC6328161 30629651

[30] Munisi DZ, Nyundo AA, Mpondo BC. Knowledge, attitude and practice towards malaria among symptomatic patients attending Tumbi Referral Hospital: A cross-sectional study. PLoS One. 2019;14(8):e0220501. 10.1371/journal.pone.0220501 31381600 PMC6681959

[31] Kala Chouakeu NA, Ngingahi LG, Bamou R, et al. Knowledge, attitude, and practices (KAP) of human populations towards malaria control in four ecoepidemiological settings in Cameroon. J Trop Med. 2021;2021(1):9925135. 10.1155/2021/9925135 34221028 PMC8213476

[32] Ng’ang’a PN, Mutunga J, Oliech G, Mutero CM. Community knowledge and perceptions on malaria prevention and house screening in Nyabondo, Western Kenya. BMC Public Health. 2019;19(1):423. 10.1186/s12889-019-6723-3 31014321 PMC6480882

